# Brain entropy changes in classical trigeminal neuralgia

**DOI:** 10.3389/fneur.2023.1273336

**Published:** 2023-11-23

**Authors:** Xiang Liu, Xiuhong Ge, Xue Tang, Haiqi Ye, Lei Pan, Xiaofen Zhu, Hanjun Hu, Zhongxiang Ding, Luoyu Wang

**Affiliations:** ^1^Department of Radiology, Hangzhou First People's Hospital, Hangzhou, Zhejiang, China; ^2^School of Medical Imaging, Hangzhou Medical College, Hangzhou, Zhejiang, China; ^3^The Fourth Clinical College, Zhejiang Chinses Medical University, Hangzhou, Zhejiang, China

**Keywords:** classical trigeminal neuralgia, resting-state functional magnetic resonance imaging, brain entropy, machine learning, cross-validation

## Abstract

**Background:**

Classical trigeminal neuralgia (CTN) is a common and severe chronic neuropathic facial pain disorder. The pathological mechanisms of CTN are not fully understood. Recent studies have shown that resting-state functional magnetic resonance imaging (rs-fMRI) could provide insights into the functional changes of CTN patients and the complexity of neural processes. However, the precise spatial pattern of complexity changes in CTN patients is still unclear. This study is designed to explore the spatial distribution of complexity alterations in CTN patients using brain entropy (BEN).

**Methods:**

A total of 85 CTN patients and 79 age- and sex-matched healthy controls (HCs) were enrolled in this study. All participants underwent rs-fMRI and neuropsychological evaluations. BEN changes were analyzed to observe the spatial distribution of CTN patient complexity, as well as the relationship between these changes and clinical variables. Sixteen different machine learning methods were employed to classify the CTN patients from the HCs, and the best-performing method was selected.

**Results:**

Compared with HCs, CTN patients exhibited increased BEN in the thalamus and brainstem, and decreased BEN in the inferior semilunar lobule. Further analyses revealed a low positive correlation between the average BEN values of the thalamus and neuropsychological assessments. Among the 16 machine learning methods, the Conditional Mutual Information Maximization-Random Forest (CMIM-RF) method yielded the highest area under the curve (AUC) of 0.801.

**Conclusions:**

Our study demonstrated that BEN changes in the thalamus and pons and inferior semilunar lobule were associated with CTN and machine learning methods could effectively classify CTN patients and HCs based on BEN changes. Our findings may provide new insights into the neuropathological mechanisms of CTN and have implications for the diagnosis and treatment of CTN.

## 1 Introduction

Classical trigeminal neuralgia (CTN) is a prevalent and debilitating chronic neuropathic facial pain disorder (1). This condition is characterized by sudden and intense facial pain episodes, often triggered by seemingly harmless activities like eating, talking, or teeth brushing (2, 3). CTN significantly impacts the quality of life and daily functioning of those affected, highlighting the need for a deeper comprehension of its underlying pathological mechanisms, however, its precise pathological mechanisms remain elusive (4, 5). The current understanding suggests that CTN arises from neurovascular compression, which leads to demyelination and ectopic neuronal firing in the trigeminal nerve (2, 6–8). However, this explanation fails to capture the complexity and heterogeneity of the disorder, indicating the involvement of additional factors in the development and maintenance of CTN (9–14).

In recent years, resting-state functional magnetic resonance imaging (rs-fMRI) has emerged as a valuable tool for investigating the functional changes associated with neurological disorders (15, 16). By measuring blood oxygenation level-dependent (BOLD) signals, rs-fMRI allows the assessment of neural activity and connectivity in the brain (17, 18). Several studies have utilized rs-fMRI to explore the functional alterations in CTN patients and gain insights into the complex neural processes underlying the disorder (12, 19, 20). Recent research findings indicate dynamic changes in the static and dynamic amplitude of low-frequency fluctuation (ALFF) within 5 seconds and 30 min (19). Similar results have been found in studies examining static and dynamic degree centrality (20).

Despite advances in rs-fMRI studies on CTN, the spatial distribution of complexity changes in CTN patients is still limited. Brain entropy (BEN), which is derived from rs-fMRI data, has proven to be a valuable tool for mapping temporal signal complexity throughout the entire brain (21). BEN exhibits unique features for assessing brain function compared to fractional ALFF and cerebral blood flow (22). Recent studies have demonstrated the neurocognitive correlates of resting BEN in the default mode network and executive control network (23) and reported that lower resting brain entropy is associated with stronger task activation and deactivation in various tasks (24). It is worth noting that alterations in BEN have been observed in caffeine ingestion (25), repetitive transcranial magnetic stimulation (26) and various brain disorders, including Alzheimer's disease (27), autism spectrum disorder (28), major depressive disorder (29), and manic and euthymic adolescent bipolar disorder (30). These studies have compellingly showcased the unique role of BEN in detecting both normal brain function and various brain disorders. However, the role of BEN in CTN patients remains unknown.

This study aims to investigate the spatial distribution of complexity changes in CTN patients. By comparing CTN patients to healthy controls (HCs), we aimed to identify regions exhibiting altered complexity and explore their functional implications. By examining the patterns of BEN alterations, we hope to gain a better understanding of the neural mechanisms underlying CTN and identify potential biomarkers for disease diagnosis and treatment evaluation.

## 2 Materials and methods

### 2.1 Participants

Between July 2021 and March 2022, a total of 85 patients diagnosed with CTN and 79 age- and sex-matched HCs were prospectively recruited from the Department of Radiology at Hangzhou First People's Hospital, Zhejiang University School of Medicine. Written informed consent was obtained from all participants prior to their involvement in this prospective study. The study was conducted in accordance with the ethical guidelines and principles outlined in the Declaration of Helsinki and was approved by the local ethics committee of Hangzhou First People's Hospital, Zhejiang University School of Medicine (IRB# No. 202107002).

The inclusion criteria for CTN patients were as follows: (1) diagnosis of CTN based on the third edition of the International Classification of Headache Disorders (ICHD-3), with magnetic resonance imaging (MRI) evidence of neurovascular compression (NVC) showing morphological changes (atrophy or dislocation) in the trigeminal nerve root (1); (2) unilateral pain in the distribution of one or more branches of the trigeminal nerve; (3) paroxysmal facial pain triggered by specific factors (e.g., light touching of the face, opening of the mouth, etc.); (4) normal brain signals on conventional MRI T1WI and T2WI scans; (5) no additional neurological or sensory deficits; (6) no prior surgical or invasive procedures for CTN; (7) no contraindications to MRI; (8) right-handedness; and (9) all patients underwent microvascular decompression, confirming the presence of NVC rather than just contact.

The exclusion criteria were as follows: (1) CTN patients who had undergone previous surgical treatment; (2) presence of headaches or other paroxysmal or chronic pain conditions; (3) family history of headache or other pain in first-degree relatives; (4) presence of other somatic or psychiatric conditions; (5) left-handedness; and (6) contraindications to MRI.

### 2.2 Multimodal MRI data acquisition

All participants underwent MRI scans using a 3.0 T MRI scanner (Siemens, MAGNETOM Verio, Germany) equipped with an eight-channel phased-array head coil. The structural data were acquired using 3D T1 weighted images (magnetization-prepared rapid gradient-echo) and the functional images were acquired using gradient echo-echo planar imaging. The imaging parameters were as follows: for 3D T1 weighted images, 176 slices were acquired with a repetition time (TR) of 1,900 ms, an echo time (TE) of 2.52 ms, a slice thickness of 1 mm, a field of view (FOV) of 256 × 256 mm^2^, a voxel size of 1 × 1 × 1 mm^3^, and a flip angle of 9 degrees. For functional images, 240 volumes were acquired with a TR of 2,000 ms, a TE of 30 ms, a slice thickness of 3.2 mm, a voxel size of 3.44 × 3.44 × 3.20 mm^3^, a flip angle of 90 degrees, a FOV of 220 × 220 mm^2^, and a scan duration of 8 min. Participants were instructed to keep their eyes closed, remain awake, and breathe calmly throughout the scan, controlled breathing throughout the scan to ensure they were in a resting state.

### 2.3 Data preprocessing

The rs-fMRI data preprocessing was performed using the DPABI and SPM12 toolbox in MATLAB (MathWorks, MA, USA). The preprocessing pipeline involved the following steps: (1) discarding the initial 10 volumes to achieve a steady-state MRI signal; (2) correcting for slice timing and head motion using motion correction parameters; (3) co-register the structural MRI image (T1-weighted) to the mean functional image created after motion correction; (4) the T1-weighted structural images were then segmented and normalized to the Montreal Neurological Institute (MNI) space to obtain deformation information using Diffeomorphic Anatomical Registration Through Exponentiated Lie Algebra (DARTEL); (5) normalizing the images to the MNI space by deformation fields derived from tissue segmentation of structural images as described above and resampling to a voxel size of 3 × 3 × 3 mm^3^; (6) detrending the BOLD signal by removing the linear trend; (7) removing noise through regression of Friston-24 head motion parameters, cerebrospinal fluid signals, and white matter signals; and (8) band-pass filtering was applied in 0.01–0.08 Hz. Nine patients and seven HCs were excluded due to excessive head motion (displacement > 3 mm, rotation > 3°) or framewise displacement (FD_Jenkinson) > 0.2. The final analysis included 76 patients with CTN and 72 HCs.

### 2.4 Brain entropy analysis

After image pre-processing, the calculation of BEN was performed using BENtbx software (21), which can be accessed at https://cfn.upenn.edu/~zewang/software.html. BEN was computed at each voxel using Sample Entropy (SampEn) (31). SampEn is an approximate entropy measure that quantifies the temporal coherence of a time series by assessing the likelihood that a small section of the data matches with other sections as the window length increases. The matching criterion is determined by a threshold set as a multiple (r) of the standard deviation of the entire time series. In this study, a window length of 3 and a cutoff threshold of 0.6 times the standard deviation were used (21). Further information on BEN calculation can be found in the original BENtbx paper (21). The resulting entropy values of all voxels constituted the BEN map, which was then smoothed with an isotropic Gaussian kernel (full width at half maxima = 6 mm) to mitigate structural brain differences among individuals that may arise during spatial normalization to the MNI space.

### 2.5 Statistical analysis

For demographic and clinical continuous variables, normality was assessed using the Shapiro-Wilk test. Normally distributed variables were reported as mean ± standard deviation, and group comparisons were conducted using two-sample *t-*tests. Non-normally distributed variables were reported as median (interquartile range), and group comparisons were performed using the Mann-Whitney U test. Gender differences between groups were examined using the chi-square test.

In the DPABI software, differences in brain entropy between patients and HCs were assessed using a two sample *t-*test, and then multiple comparison correction based on Gauss random field (GRF) theory was applied with significance thresholds set at voxel-wise *p* < 0.001 and cluster-wise *p* < 0.05. To account for potential confounding factors, such as age, sex, education, and head motion, these variables were included as covariates in the analysis. Additionally, partial correlation analysis was performed to explore the associations between the average BEN values of significant brain areas and the neuropsychological assessments, regressing out the covariates of age, sex, education, and head motion.

### 2.6 Machine learning classification

Machine learning was conducted using a script within the MATLAB environment. In our study, we utilized the Automated Anatomical Labeling (AAL) atlas version 3 as a mask, and for each voxel, we extracted the corresponding BEN value as a feature. This process resulted in a total of 54,948 MRI features, each corresponding to a specific voxel defined by the AAL atlas. Previous studies have demonstrated the reliability and reproducibility of the temporal complexity of rs-fMRI (32).

Considering the limited availability of subjects in human neuroimaging studies, which can impact the generalization ability of the study, we employed a 10-fold cross-validation strategy. In each iteration of cross-validation, one-tenth of the samples were held out as a test set, while the remaining subjects were used for training the classifier. This process was repeated until all subjects were used as test samples. Within each fold of cross-validation, we first performed a two-sample *t-*test between the two groups to remove features with *p* > 0.001 and then utilized four widely recognized feature selection methods based on filter approaches: fisher score (FSCR), relief-F (RELF-F), minimum redundancy maximum relevance (MRMR), and conditional mutual information maximization (CMIM). To address redundancy, features exhibiting a Spearman's correlation of 0.7 or higher were eliminated to reduce the presence of redundant information.

Moreover, in our effort to address the challenges associated with the “curse of dimensionality” and mitigate the risk of overfitting, we followed Harrell's guideline, which recommends that the number of selected features should be <10% of the sample size, with a final feature count of approximately 10 (33–35). Consequently, we selected the top 10 non-redundant features (36, 37) as the input for four distinct machine learning classifiers: logistic regression (LR), k-nearest neighbor (KNN), random forest (RF), and support vector machines with radial basis function kernel (RBF-SVM). In response to the question of whether selecting more or fewer features would significantly improve classification performance, we conducted additional experiments. We validated the results using the first 5 features and the first 20 features, respectively. The results for these different feature selection scenarios were analyzed and compared to the results obtained using the top 10 features. To ensure the generalizability of the biomarkers and avoid overfitting and spurious results (38), we utilized standard/default parameter settings for all experiments, rather than extensive hyperparameter tuning, a choice consistent with the previous study (39). We implemented 16 cross-combinations of feature selection methods and classifiers (4 feature selection methods × 4 classifiers). Finally, the results of each repetition were averaged to produce the final classification outcome.

To further validate the significance of our classification results, we conducted permutation tests on classification metrics (accuracy, sensitivity, specificity and area under the curve [AUC]). In these permutation tests, we generated four null distributions of classification metrics (AUC, accuracy, sensitivity and specificity) for each classifier using a non-parametric permutation approach with 2,000 permutations. The *p*-value was determined by calculating the proportion of the 2,000 permutations for which the classification metrics of the original data were equal to or larger than the classification metrics of the permuted data. The classification metrics of each classifier were considered statistically significant if the *p*-values were <5% (*p* < 0.05).

## 3 Results

### 3.1 Demographics and clinical characteristics

Demographic and clinical information for the final 76 CTN patients [56 (13) years, 52 females] and 72 HCs [54.5 (14) years, 47 females] were presented in Table 1 and Supplementary Table 1. There were no significant differences in gender (*p* = 0.685), age (*p* = 0.066), and head motion (*p* = 0.954), and while significant differences were observed in Mini-Mental State Examination (MMSE, *p* < 0.001), the quality-of-life score of patients with trigeminal neuralgia (*p* < 0.001), self-rating depression scale (SDS, *p* < 0.001), self-rating anxiety scale (SAS, *p* < 0.001) between the two groups.

**Table 1:** Demographic and clinical characteristics of CTN patients and HCs.

	**CTN**	**HCs**	***Z|χ^2^***	***P***
Gender (female:male)	52:24	47:25	0.165	0.685^a^
Age [years, M (IQR)]	56.000 (13.000)	54.500 (14.000)	−1.840	0.066^b^
Education [years, M (IQR)]	9.000 (3.000)	10.000 (6.000)	−3.641	0.000^b^
MMSE	27.500 (4.000)	29.000 (2.000)	−3.999	0.000^b^
Symptoms^*^	22.000 (6.000)	6.000 (0.000)	−11.164	0.000^b^
Physical function^*^	19.000 (6.000)	14.000 (5.000)	−7.250	0.000^b^
Psychology^*^	12.500 (6.000)	5.000 (0.000)	−9.962	0.000^b^
Society^*^	14.000 (6.000)	5.000 (0.000)	−11.047	0.000^b^
Total	69.000 (15.000)	31.000 (5.000)	−10.502	0.000^b^
Self-rating depression scale	38.000 (11.000)	28.000 (8.000)	−7.000	0.000^b^
Self-rating anxiety scale	33.000 (8.000)	27.000 (7.000)	−5.184	0.000^b^
Head motion (FD)	0.057 (0.044)	0.060 (0.049)	−0.058	0.954^b^

a *p*-values for sex distribution obtained by the chi-square test;

b *p-*value obtained by the Wilcoxon Mann Whitney test. CTN, classical trigeminal neuralgia; HCs, healthy controls;

* the four dimensions of the quality of life score of patients with trigeminal neuralgia; R, right; L, left; MMSE, mini-mental state examination.

### 3.2 Spatial distribution of BEN changes and correlation analysis

**Figure 1:**
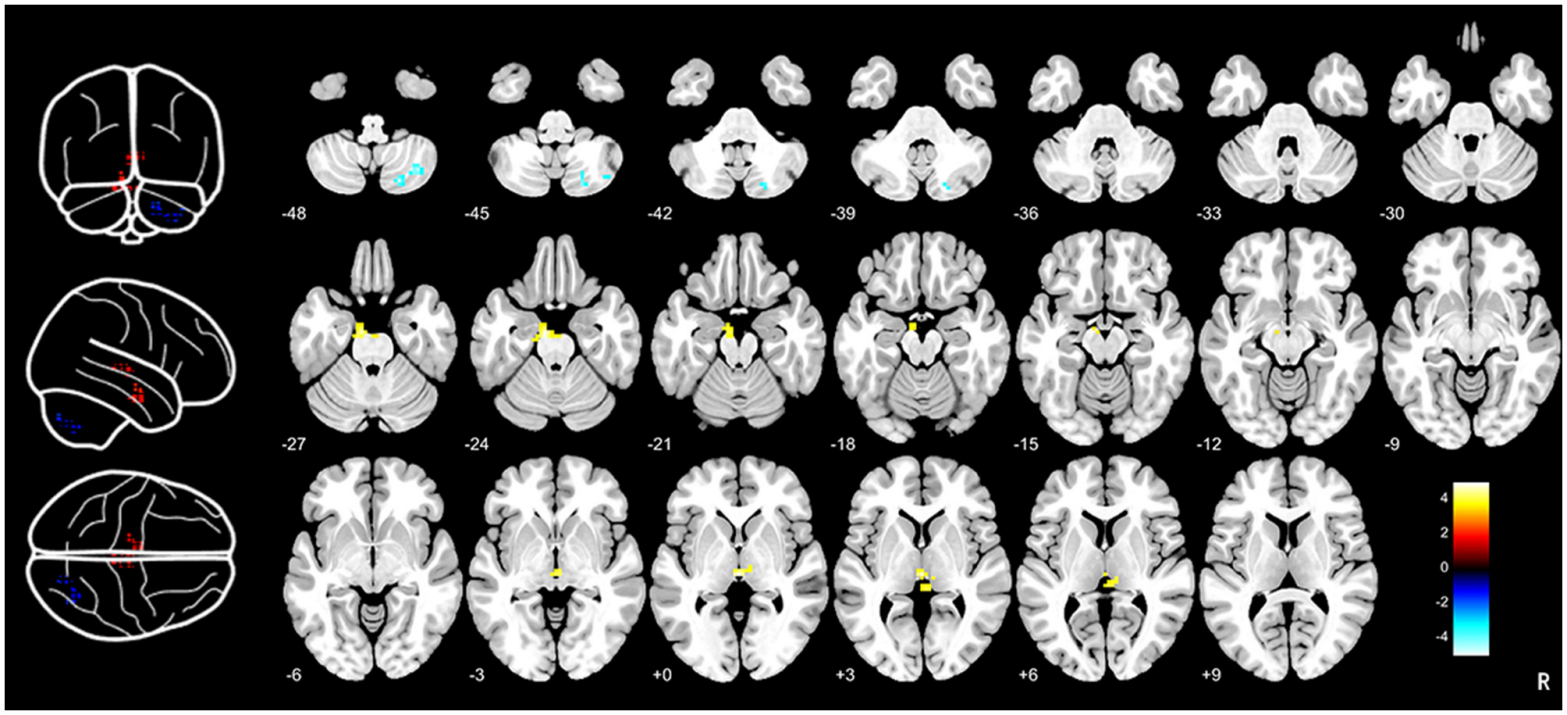
Brain entropy analysis between CTN patients and HCs. Brain entropy map showed that significant differences in thalamus, pons and inferior semilunar lobule between CTN patients and HCs. Increased BEN was observed in the thalamus (voxel *p* < 0.001, cluster *p* < 0.05, GRF correction, cluster size > 32 voxels) and pons (voxel *p* < 0.001, cluster *p* < 0.05, GRF correction, cluster size > 32 voxels) and decreased BEN was found in the inferior semilunar lobule (voxel *p* < 0.001, cluster *p* < 0.05, GRF correction, cluster size > 32 voxels). CTN, classical trigeminal neuralgia; HCs, healthy controls.

**Figure 2:**
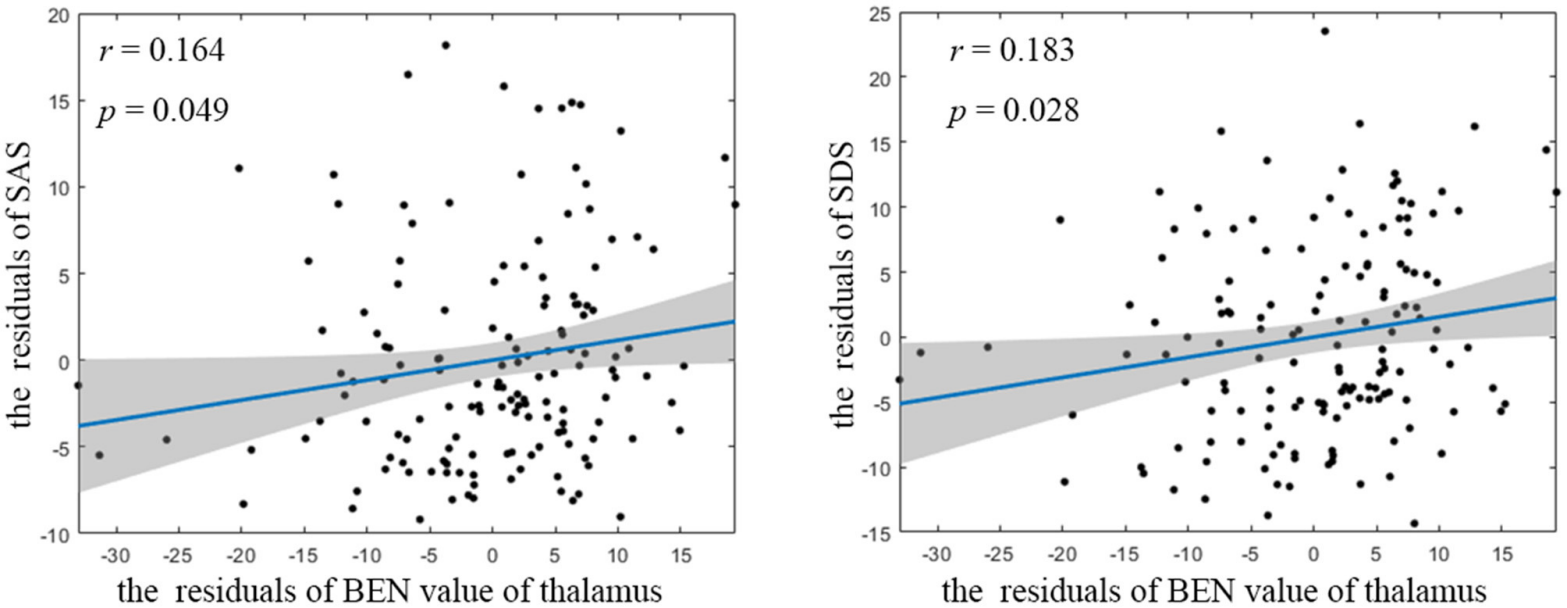
Partial correlation between BEN value of thalamus and neuropsychological assessment. BEN, brain entropy; SAS, self-rating anxiety scale; SDS, self-rating depression scale.

Compared to HCs, CTN patients exhibited distinct patterns of BEN changes in the brain (Table 2, Figure 1). Specifically, increased BEN was observed in the thalamus (voxel *p* < 0.001, cluster *p* < 0.05, GRF correction, cluster size > 32 voxels) and pons (voxel *p* < 0.001, cluster *p* < 0.05, GRF correction, cluster size > 32 voxels), suggesting enhanced neural activity and complexity in these areas. Conversely, decreased BEN was found in the inferior semilunar lobule (voxel *p* < 0.001, cluster *p* < 0.05, GRF correction, cluster size > 32 voxels), indicating disrupted neural processing and complexity in this region. Further analyses revealed a low positive correlation between the average BEN values of the thalamus and neuropsychological assessments (SAS and SDS), regressing out the covariates of age, sex, education, and head motion (Figure 2, Table 3).

**Table 2:** Brain regions with significant differences in BEN between CTN patients and HCs (GRF correction, voxel level *p* < 0.001 and cluster level *p* < 0.05).

**Brain region**	**Peak MNI coordinates**			**Cluster size (voxels)**	**Peak intensity t**	***p-value***
	**X**	**Y**	**Z**			
Thalamus	0	−21	0	35	4.8069	< 0.001
Pons	9	−15	−24	44	4.8286	< 0.001
Right inferior semilunar lobule	33	−69	−51	43	−5.1713	< 0.001

MNI, Montreal Neurological Institute; CTN, classical trigeminal neuralgia; HCs, healthy controls.

**Table 3:** Partial correlation between BEN value of each significant cluster and neuropsychological assessment.

**Brain regions**	**MMSE**		**SAS**		**SDS**	
	**r**	***p*** **-value**	**r**	***p*** **-value**	**r**	***p*** **-value**
Thalamus	−0.015	0.856	0.164	0.049^*^	0.183	0.028^*^
Pons	−0.019	0.825	0.099	0.239	0.150	0.072
Right inferior semilunar lobule	0.053	0.525	−0.014	0.869	−0.156	0.062

MMSE, mini mental state test; SAS self-rating anxiety scale; SDS, self-rating depression scale;

* represents significant level *p* < 0.05.

### 3.3 Machine learning classification

**Figure 3:**
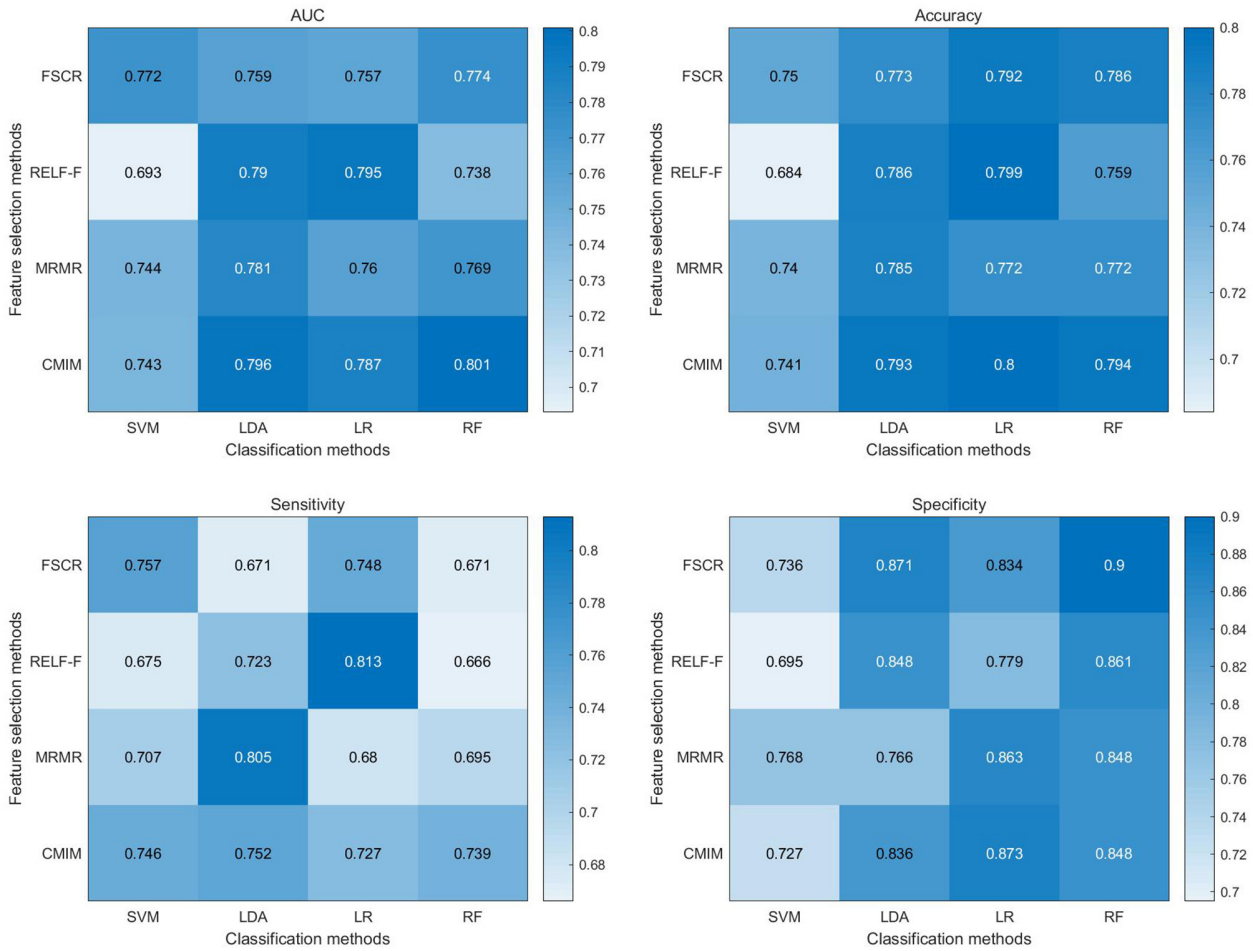
Heat map depicting diagnostic performance (AUC, accuracy, sensitivity, specificity) of paired feature selection (rows) and classification (columns) methods. FSCR, fisher score; RELF-F, relief-F; MRMR, minimum redundancy maximum relevance; CMIM, conditional mutual information maximization; LR, logistic regression; KNN, k-nearest neighbor; RF, random forest; RBF-SVM, support vector machines with radial basis function kernel; AUC, area under the curve.

The classification metrics, including AUC, accuracy, sensitivity, and specificity, are presented in Figure 3. Among the 16 combinations of machine learning methods utilized in our study, the CMIM-RF method demonstrated the highest performance in classifying CTN patients and HCs based on BEN changes, achieving an AUC of 0.801, and the AUC of most classifiers above 0.75.

Supplementary Tables 2–5 presents the results of permutation tests for classification metrics. The outcomes of these permutation tests consistently revealed statistical significance (*p* < 0.05) across all classification metrics of CMIM-RF, including accuracy, sensitivity, specificity, and AUC. The accuracy and AUC of the other classifiers were also statistically significant (*p* < 0.05). These results provide robust evidence that the pattern of complexity alterations captured by BEN can effectively distinguish CTN patients from HCs, further underscoring its potential as a diagnostic tool for CTN.

Moreover, it is noteworthy that our findings remain stable across different feature selection scenarios. Specifically, the results obtained using the first 5 features and the first 20 features, as illustrated in Supplementary Figures 1, 2, exhibited similar performance, reinforcing the reliability, and consistency of our findings.

## 4 Discussion

The present study investigated the spatial distribution of BEN changes in CTN patients using rs-fMRI. Our findings revealed distinct patterns of complexity alterations in key brain regions associated with the experience of pain and the psychological aspects. The increased BEN was observed in the thalamus and brainstem, while the BEN decreased in the inferior semilunar lobule. In addition, the results of the correlation showed a positive correlation between BEN changes in the thalamus and brainstem regions. Among the 16 machine learning methods tested, the CMIM-RF method showed the highest performance in classifying CTN patients and HCs based on BEN changes, with an AUC of 0.801, highlighting its potential as a diagnostic tool for CTN.

CTN patients exhibited distinct patterns of complexity changes in the brain, with increased BEN observed in the thalamus and pons regions, indicating enhanced neural activity and complexity (21). Consistent with this, a multimodal MRI study by Wang et al. revealed increased mean diffusivity in the thalamus and brainstem of individuals with CTN (9). The thalamus is known to be a key relay station for pain signals, and its hyperactivity has been implicated in various chronic pain disorders (40, 41). The increased complexity in the thalamus suggests altered information processing and integration, which may contribute to the generation and maintenance of facial pain in CTN. Ge et al. (19) conducted an rs-fMRI study on pain induced by stimulation and found that in CTN patients, the values of the right thalamus decreased immediately after the triggering event and subsequently increased within 30 min. This suggests dynamic changes in thalamic activity and highlights its involvement in the pathophysiology of CTN (19). The brainstem, on the other hand, plays a vital role in pain modulation and descending pain inhibition (42, 43). The enhanced complexity in the brainstem could indicate abnormal pain modulation mechanisms, leading to an imbalance between pain facilitation and inhibition processes in CTN.

The decreased complexity observed in the inferior semilunar lobule is an interesting finding that warrants further investigation. This region is part of the cerebellum, which is traditionally associated with motor coordination but has also been implicated in pain processing (44, 45). Previous studies have demonstrated that the cerebellum plays a role in pain modulation and the integration of sensory information (46, 47). The disrupted BEN in the inferior semilunar lobule suggests an alteration in its functional complexity and information processing, which may contribute to abnormal pain perception and sensory integration in CTN (48). Further investigation is needed to elucidate the underlying mechanisms and functional implications of this finding.

The observed positive correlation between the average BEN values of the thalamus and neuropsychological assessments (SAS and SDS) further supports the notion that the thalamus plays a crucial role in the pathophysiology of CTN, suggesting a potential relationship between the complexity of brain activity in the thalamus and the psychological wellbeing of these patients (49). This correlation implies that as the BEN values in the thalamus increase (indicating higher neural complexity), there is a tendency for higher scores on anxiety and depression scales. In summary, the provided evidence suggests that distinct complexity alterations in key brain regions, particularly the thalamus, are associated with the experience of pain and the psychological aspects of CTN.

The machine learning classification analysis demonstrated the potential utility of BEN changes as a diagnostic marker for CTN. CMIM is an effective feature selection algorithm that considers both relevance and redundancy among features (50). It selects features that are highly informative for classification while minimizing the redundancy between them. This helps to improve the efficiency and accuracy of the classification model by focusing on the most relevant and non-redundant features. CMIM exhibited higher diagnostic performance in combination with the majority of classifiers. RF is a powerful and widely used classification algorithm that often shines when dealing with complex, high-dimensional data, and when robustness to overfitting is crucial (51). By integrating CMIM with RF, the CMIM-RF method capitalizes on the strengths of both algorithms, leading to significantly improved classification performance. In our study, the CMIM-RF method exhibited the highest classification performance with an AUC of 0.801 (*p* < 0.05). Moreover, most classifiers achieved AUCs above 0.75 (*p* < 0.05), underscoring the effectiveness of BEN in distinguishing CTN patients from HCs based on complexity alterations in brain activity. Notably, when considering the use of the first 5 or first 20 features instead of the top 10 features, we observed similar classification performance. This indicates that while the initial choice of the top 10 features was based on their rankings, the classification performance remained robust when varying the number of selected features within this range. These compelling findings suggest that BEN analysis holds promise as an objective and non-invasive biomarker for CTN, offering valuable support for its diagnosis and monitoring.

However, there are some limitations to consider. Firstly, the sample size of the study might have been relatively small, which could limit the generalizability of the findings. A larger and more diverse sample would provide a stronger basis for concluding the spatial distribution of complexity changes. Additionally, the cross-sectional design of the study precludes the establishment of causal relationships between BEN changes and CTN. Future longitudinal studies are needed to elucidate the temporal dynamics and potential predictive value of BEN alterations in CTN.

In conclusion, our study contributes to the understanding of CTN by revealing the spatial distribution of complexity changes in the brains of CTN patients. The increased complexity in the thalamus and brainstem, along with the decreased complexity in the inferior semilunar lobule, suggests altered neural processing and complexity in key regions implicated in pain perception and modulation. The machine learning classification analysis highlights the potential of BEN changes as a diagnostic marker for CTN. These findings provide new insights into the neuropathological mechanisms of CTN and may have implications for the diagnosis, treatment, and monitoring of this debilitating facial pain disorder.

## Data availability statement

The raw data supporting the conclusions of this article will be made available by the authors, without undue reservation.

## Ethics statement

The study was approved by the Local Ethics Committee of Hangzhou First People's Hospital, Zhejiang University School of Medicine (IRB# No. 202107002). The studies were conducted in accordance with the local legislation and institutional requirements. The participants provided their written informed consent to participate in this study.

## Author contributions

XL: Writing—original draft, Writing—review & editing, Data curation. XG: Data curation, Writing—review & editing. XT: Visualization, Writing—review & editing. HY: Data curation, Writing—review & editing. LP: Data curation, Writing—review & editing. XZ: Data curation, Writing—review & editing. HH: Visualization, Writing—review & editing. ZD: Conceptualization, Funding acquisition, Project administration, Resources, Writing—review & editing. LW: Conceptualization, Methodology, Writing—original draft, Writing—review & editing.
