# Supplementary material for: Brain entropy changes in classical trigeminal neuralgia
Source: Front Neurol. 2023 Nov 23;14:1273336. doi: 10.3389/fneur.2023.1273336 (PMC10701740; doi:10.3389/fneur.2023.1273336)
Supplement: Supplementary file 1 [file Data_Sheet_1.docx]

Supplementary Material

## Supplementary Figures


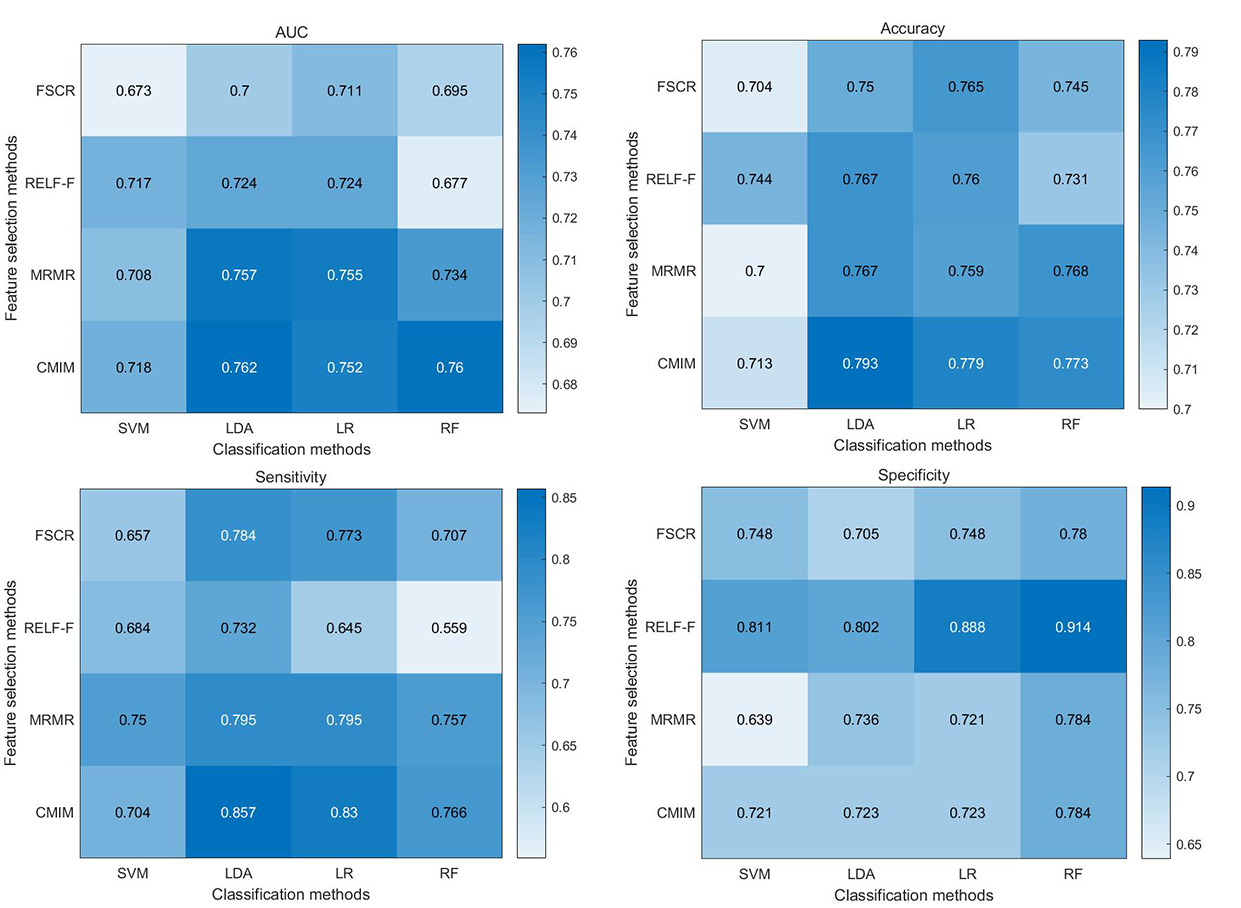


**Supplementary Figure 1.** Heat map depicting diagnostic performance (AUC, accuracy, sensitivity, specificity) of paired feature selection (rows) and classification (columns) methods for the first 5 features.

*FSCR, fisher score; RELF-F, relief-F; MRMR, minimum redundancy maximum relevance; CMIM, conditional mutual information maximization; LR, logistic regression; KNN, k-nearest neighbor; RF, random forest; RBF-SVM, support vector machines with radial basis function kernel.*


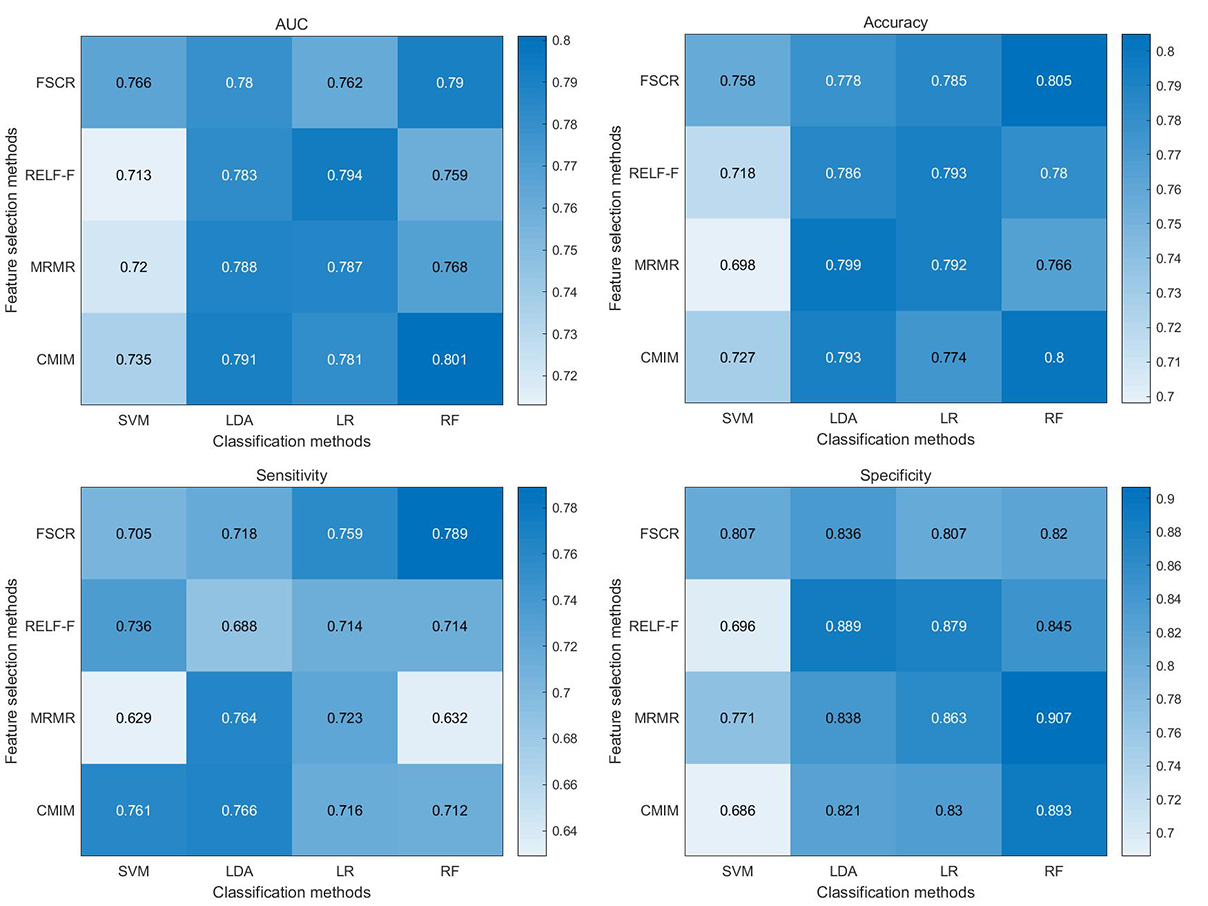


**Supplementary Figure 2.** Heat map depicting diagnostic performance (AUC, accuracy, sensitivity, specificity) of paired feature selection (rows) and classification (columns) methods for the first 20 features.

*FSCR, fisher score; RELF-F, relief-F; MRMR, minimum redundancy maximum relevance; CMIM, conditional mutual information maximization; LR, logistic regression; KNN, k-nearest neighbor; RF, random forest; RBF-SVM, support vector machines with radial basis function kernel.*

|  | Brain region | Age | | |
| --- | --- | --- | --- | --- |
|  |  | *r* | *p* |  |
| CTN | thalamus | -0.219 | 0.064 |  |
|  | pons | -0.184 | 0.121 |  |
|  | right inferior semilunar lobule | 0.039 | 0.748 |  |
| HC | thalamus | -0.029 | 0.806 |  |
|  | pons | -0.013 | 0.913 |  |
|  | right inferior semilunar lobule | -0.146 | 0.208 |  |

**Supplementary Table 1.** Correlation between BEN values in three specific brain regions and age.

*BEN, Brain entropy; CTN, classical trigeminal neuralgia; HCs, healthy controls.*

| AUC (*p*) | SVM | LDA | LR | RF |
| --- | --- | --- | --- | --- |
| FSCR | <0.001 | <0.001 | <0.001 | <0.001 |
| RELF-F | <0.001 | <0.001 | <0.001 | <0.001 |
| MRMR | <0.001 | <0.001 | <0.001 | <0.001 |
| CMIM | <0.001 | <0.001 | <0.001 | <0.001 |

**Supplementary Table 2. Results of permutation test for AUC.**

*FSCR, fisher score; RELF-F, relief-F; MRMR, minimum redundancy maximum relevance; CMIM, conditional mutual information maximization; LR, logistic regression; KNN, k-nearest neighbor; RF, random forest; RBF-SVM, support vector machines with radial basis function kernel;* AUC, *area under the curve.*

| accuracy (*p*) | SVM | LDA | LR | RF |
| --- | --- | --- | --- | --- |
| FSCR | <0.001 | <0.001 | <0.001 | <0.001 |
| RELF-F | <0.001 | <0.001 | <0.001 | <0.001 |
| MRMR | <0.001 | <0.001 | <0.001 | <0.001 |
| CMIM | <0.001 | <0.001 | <0.001 | <0.001 |

**Supplementary Table 3. Results of permutation test for accuracy.**

*FSCR, fisher score; RELF-F, relief-F; MRMR, minimum redundancy maximum relevance; CMIM, conditional mutual information maximization; LR, logistic regression; KNN, k-nearest neighbor; RF, random forest; RBF-SVM, support vector machines with radial basis function kernel.*

| sensitivity (*p*) | SVM | LDA | LR | RF |
| --- | --- | --- | --- | --- |
| FSCR | 0.005 | 0.197 | 0.046 | 0.190 |
| RELF-F | 0.022 | 0.084 | 0.011 | 0.198 |
| MRMR | 0.011 | 0.0080 | 0.152 | 0.118 |
| CMIM | 0.003 | 0.043 | 0.066 | 0.041 |

**Supplementary Table 4. Results of permutation test for sensitivity.**

*FSCR, fisher score; RELF-F, relief-F; MRMR, minimum redundancy maximum relevance; CMIM, conditional mutual information maximization; LR, logistic regression; KNN, k-nearest neighbor; RF, random forest; RBF-SVM, support vector machines with radial basis function kernel.*

| specificity (*p*) | SVM | LDA | LR | RF |
| --- | --- | --- | --- | --- |
| FSCR | 0.519 | 0.021 | 0.056 | 0.007 |
| RELF-F | 0.696 | 0.037 | 0.187 | 0.032 |
| MRMR | 0.384 | 0.243 | 0.026 | 0.059 |
| CMIM | 0.558 | 0.058 | 0.023 | 0.046 |

**Supplementary Table 5. Results of permutation test for specificity.**

*FSCR, fisher score; RELF-F, relief-F; MRMR, minimum redundancy maximum relevance; CMIM, conditional mutual information maximization; LR, logistic regression; KNN, k-nearest neighbor; RF, random forest; RBF-SVM, support vector machines with radial basis function kernel.*
